# Supplementary material for: Molecular Characterization Analysis and Adaptive Responses of Spodoptera frugiperda (Lepidoptera: Noctuidae) to Nutritional and Enzymatic Variabilities in Various Maize Cultivars
Source: Plants (Basel). 2024 Feb 22;13(5):597. doi: 10.3390/plants13050597 (PMC10934461; doi:10.3390/plants13050597)
Supplement: Supplementary file 1 [file plants-13-00597-s001.zip › plants-2836884-supplementary.pdf]

Supplementary Table S1. Content analysis of plant nutrients and secondary substances in six maize varieties

Maize variety	Protein (mg/g)	Amino acids (umol/g)	Fatty acid (umol/g)	Reducing sugar(mg/g)	Tannin (nmol/g)	Flavone (mg/g)
Zhengdan 958	(17.2±0.2) a	(119.1±21.3) a	(0.7±0.3) c	(6.8±0.1) bc	(6.3±0.9) d	(0.9±0.1) bc
Wuke No.4	(10.5±1.1) c	(87.9±7.0) b	(0.6±0.2) c	(7.4±0.7) b	(5.1±1.1) d	(1.1±0.1) ab
Wuke 113	(15.7±0.7) ab	(82.3±7.0) bc	(0.9±0.3) c	(6.7±0.4) c	(10.6±0.9) a	(0.9±0.2) bc
Zaocuiwang	(13.9±2.2) b	(77.7±14.0) bc	(2.4±0.3) b	(6.9±0.1) bc	(7.7±0.2) c	(1.0±0.1) bc
Baitiannuo	(10.3±1.7) c	(54.0±20.5) cd	(2.6±0.1) ab	(7.3±0.1) bc	(9.0±0.9) bc	(0.8±0.1) c
Ziyunuo	(9.9±0.7) c	(45.6±13.5) d	(3.0±0.5) a	(8.4±0.2) a	(9.6±0.2) ab	(1.4±0.3) a

Supplementary Table S2. Analysis of water and chlorophyll content in six maize varieties

Maize variety	Moisture (%)	Chlorophyll a (mg/g)	Chlorophyll b (mg/g)	Carotenoids (mg/g)
Zhengdan 958	(1.1±0.1) ab	(1.2±0.1) ab	(0.4±0.1) a	(0.2±0) a
Wuke No.4	(1.0±0) b	(1.5±0.3) a	(0.4±0.1) a	(0.2±0.1) a
Wuke 113	(1.2±0) a	(1.0±0.2) b	(0.3±0.1) ab	(0.2±0) a
Zaocuiwang	(0.6±0.1) c	(0.9±0.1) b	(0.3±0) ab	(0.2±0) a
Baitiannuo	(1.0±0) b	(1.1±0.2) b	(0.3±0) ab	(0.2±0) a
Ziyunuo	(0.5±0) c	(1.0±0.1) b	(0.2±0.1) b	(0.2±0) a
